# Two tautomers in the same crystal: 3-(4-fluoro­phen­yl)-1*H*-pyrazole and 5-(4-fluoro­phen­yl)-1*H*-pyrazole

**DOI:** 10.1107/S160053681401695X

**Published:** 2014-08-01

**Authors:** Thammarse S. Yamuna, Manpreet Kaur, Jerry P. Jasinski, Brian J. Anderson, H. S. Yathirajan

**Affiliations:** a Department of Studies in Chemistry, University of Mysore, Manasagangotri, Mysore 570 006, India; b Department of Chemistry, Keene State College, 229 Main Street, Keene, NH 03435-2001, USA

**Keywords:** crystal structure, pyrazole derivative, tautomeric forms, hydrogen bonds

## Abstract

The title co-crystal, 3-(4-fluoro­phen­yl)-1*H*-pyrazole–5-(4-fluoro­phen­yl)-1*H*-pyrazole (1/1), C_9_H_7_FN_2_, crystallizes with four independent mol­ecules (*A*, *B*, *C* and *D*) in the asymmetric unit exhibiting two tautomeric forms (*A* and *D*; *B* and *C*) due to N—H proton exchange between the two N atoms of the pyrazole ring. The dihedral angles between the mean planes of the pyrazole and benzene rings are 15.6 (1), 19.8 (9), 14.0 (1) and 10.7 (7)° in mol­ecules *A*, *B*, *C* and *D*, respectively. In the crystal, N—H⋯N hydrogen bonds link the four mol­ecules in the asymmetric unit into a ring with an *R*_4_^4^(12) motif. Furthermore, weak C—H⋯F inter­actions link the mol­ecules into a three-dimensional network.

## Related literature   

For biological and pharmacological properties of pyrazole compounds, see: Isloor *et al.* (2009 ▶); Patel *et al.* (2010 ▶); Sarojini *et al.* (2010 ▶); Samshuddin *et al.* (2012 ▶). For related structures, see: Baktır *et al.* (2011 ▶); Fun *et al.* (2012 ▶); Yamuna *et al.* (2013 ▶). For bond-length data, see: Allen *et al.* (1987 ▶). For a description of hydrogen bonds, see: Etter *et al.* (1990 ▶).
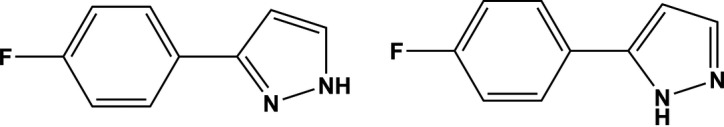


## Experimental   

### Crystal data  

C_9_H_7_FN_2_
*M* *_r_* = 162.17
Triclinic, 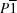

*a* = 10.3961 (5) Å
*b* = 10.8565 (6) Å
*c* = 16.1431 (7) Å
α = 84.704 (4)°
β = 76.223 (4)°
γ = 68.249 (5)°
*V* = 1643.57 (16) Å^3^
*Z* = 8
Cu *K*α radiation
μ = 0.81 mm^−1^
*T* = 173 K
0.22 × 0.16 × 0.10 mm

### Data collection  

Agilent Eos Gemini diffractometer
Absorption correction: multi-scan (*CrysAlis PRO* and *CrysAlis RED*, Agilent (2012 ▶). *T*_min_ = 0.881, *T*_max_ = 1.000
11343 measured reflections
6209 independent reflections
5042 reflections with *I* > 2σ(*I*)
*R*_int_ = 0.019

### Refinement  

*R*[*F* ^2^ > 2σ(*F* ^2^)] = 0.044
*wR*(*F* ^2^) = 0.122
*S* = 1.03
6209 reflections
449 parameters
H atoms treated by a mixture of independent and constrained refinement
Δρ_max_ = 0.26 e Å^−3^
Δρ_min_ = −0.22 e Å^−3^

### 

Data collection: *CrysAlis PRO* (Agilent, 2012 ▶); cell refinement: *CrysAlis PRO*; data reduction: *CrysAlis RED* (Agilent, 2012 ▶); program(s) used to solve structure: *SUPERFLIP* (Palatinus & Chapuis, 2007 ▶); program(s) used to refine structure: *SHELXL2012* (Sheldrick, 2008 ▶); molecular graphics: *OLEX2* (Dolomanov *et al.*, 2009 ▶); software used to prepare material for publication: *OLEX2*.

## Supplementary Material

Crystal structure: contains datablock(s) I. DOI: 10.1107/S160053681401695X/bt6989sup1.cif

Structure factors: contains datablock(s) I. DOI: 10.1107/S160053681401695X/bt6989Isup2.hkl

Supporting information file. DOI: 10.1107/S160053681401695X/bt6989Isup3.cml

**Fig. 1.**
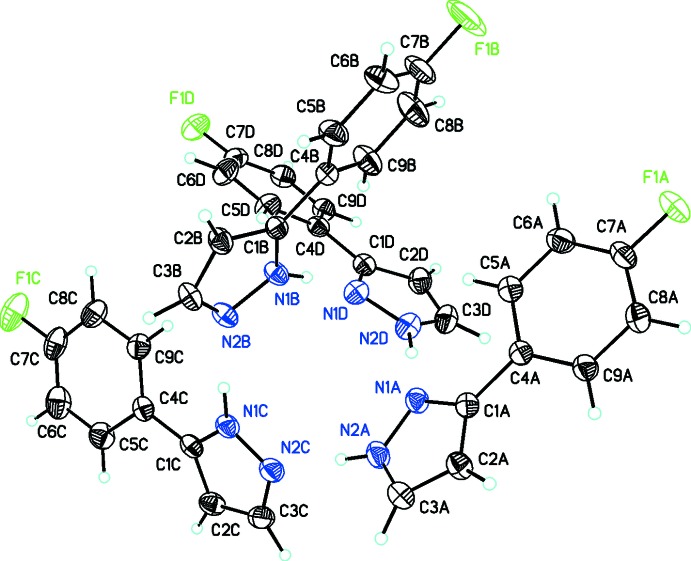
ORTEP drawing of the title compound showing the labeling scheme of the asymmetric unit of the title compound with 30% probability displacement ellipsoids.

**Fig. 2.**
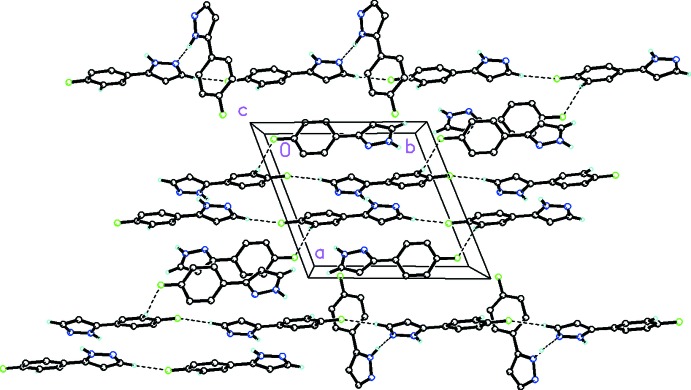
Molecular packing for the title compound viewed along the c axis. Dashed lines indicate N—H···N intermolecular hydrogen bonds and weak C—H···F intermolecular interactions together forming a 2D supramolecular network structure. H atoms not involved in hydrogen bonding have been removed for clarity.

. DOI: 10.1107/S160053681401695X/bt6989fig1.tif
ORTEP drawing of the title compound showing the labeling scheme of the asymmetric unit of the title compound with 30% probability displacement ellipsoids.

c . DOI: 10.1107/S160053681401695X/bt6989fig2.tif
Mol­ecular packing for the title compound viewed along the *c* axis. Dashed lines indicate N—H⋯N inter­molecular hydrogen bonds and weak C—H⋯F inter­molecular inter­actions together forming a 2D supra­molecular network structure. H atoms not involved in hydrogen bonding have been removed for clarity.

**Fig. 3.**
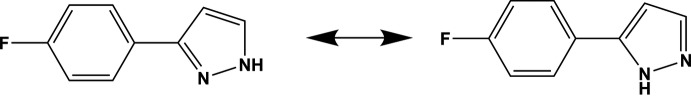
The two tautomers in the same crystal: 3-(4-fluorophenyl)-1H-pyrazole (left) and 5-(4-fluorophenyl)-1H-pyrazole (right).

H H . DOI: 10.1107/S160053681401695X/bt6989fig3.tif
The two tautomers in the same crystal: 3-(4-fluoro­phen­yl)-1*H*-pyrazole (left) and 5-(4-fluoro­phen­yl)-1*H*-pyrazole (right).

CCDC reference: 1015543

Additional supporting information:  crystallographic information; 3D view; checkCIF report

## Figures and Tables

**Table 1:** Hydrogen-bond geometry (Å, °)

*D*—H⋯*A*	*D*—H	H⋯*A*	*D*⋯*A*	*D*—H⋯*A*
N2*A*—H2*A*⋯N2*C*	0.94 (3)	1.94 (3)	2.886 (2)	177 (2)
C3*A*—H3*A*⋯F1*A* ^i^	0.95	2.58	3.226 (2)	125
N1*B*—H1*B*⋯N1*D*	1.00 (3)	1.86 (3)	2.8506 (19)	175 (2)
C3*B*—H3*B*⋯F1*B* ^i^	0.95	2.36	3.187 (2)	145
C6*B*—H6*B*⋯F1*A* ^ii^	0.95	2.51	3.287 (3)	139
N1*C*—H1*C*⋯N2*B*	0.98 (3)	1.90 (3)	2.881 (2)	173 (2)
N2*D*—H2*D*⋯N1*A*	1.02 (3)	1.87 (3)	2.896 (2)	178 (3)
C3*D*—H3*D*⋯F1*D* ^iii^	0.95	2.49	3.301 (2)	143

Symmetry codes: (i) 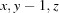; (ii) 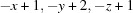; (iii) 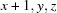.

## supplementary crystallographic information

### S1. Structural commentary

Pyrazoles are an important class of heterocyclic compounds and many pyrazole derivatives are reported to have a broad spectrum of biological properties, including anti­bacterial and anti-inflammatory activities (Patel *et al.*, 2010), anti­cancer (Sarojini *et al.*, 2010; Samshuddin *et al.*, 2012) anti-inflammatory, anti­depressant, anti­convulsant and anti-HIV properties (Isloor *et al.*, 2009). Because of these various inter­esting fields of application as well as their fairly assessable path of synthesis, the pyrazoline ring became a center of attraction for organic chemists. Crystal structures of some related compounds include 3, 5-bis­(4-fluoro­phenyl)-1-(4-nitro­phenyl)-4,5-di­hydro-1H-pyrazole (Samshuddin *et al.*, 2012), 5-(4-bromo­phenyl)- 3-(4-fluoro­phenyl)-1-phenyl-4,5-di­hydro-1H-pyrazole (Fun *et al.*, 2012), 3,5-bis­(4-fluoro­phenyl)-4,5-di­hydro-1H-pyrazole-1-carbaldehyde (Baktır *et al.*, 2011) and 3-amino-1H-pyrazol-2-ium tri­fluoro­acetate (Yamuna *et al.*, 2013). In view of the importance of the title compound, C_9_H_7_FN_2_, the paper reports its crystal structure.

The title compound, C_9_H_7_FN_2_, crystallises with four independent molecules (A,B,C and D) in the asymmetric unit exhibiting two tautomeric forms (A and D; B and C) due to N—H proton exchange between the two nitro­gen atoms (N1 and N2) of the pyrazole ring (Fig. 1). The dihedral angles between the mean planes of the pyrazole ring and phenyl ring are 15.6 (1)°, 19.8 (9)°, 14.0 (1)° and 10.7 (7)°, in the molecules A, B, C and D, respectively. Bond lengths are in normal ranges (Allen *et al.*, 1987). In the crystal, N—H···N inter­molecular hydrogen bonds link the four molecules in the asymmetric unit to a ring with motif *R*_4_^4^(12) (Etter *et al.*, 1990). Furthermore, weak C—H···F inter­molecular inter­actions link the molecules to a three-dimensional network (Fig. 2).

### S2. Synthesis and crystallization

Commercially available 3-(4-fluoro­phenyl)-1H-pyrazole was dissolved in 5 ml of di­methyl­formamide at 303 K over a heating magnetic stirrer. X-ray quality crystals were formed on slow evaporation. (m.p.: 368-373 K).

### S3. Refinement

The H atoms bonded to N (H2A, H1B, H1C and H2D) were refined isotropically and all of the remaining H atoms were placed in their calculated positions and then refined using the riding model with C—H lengths of 0.93Å. Isotropic displacement parameters for these atoms were set to 1.2 times *U*_eq_ of the parent C atom.

### Crystal data

**Table:**

C_9_H_7_FN_2_	*Z* = 8
*M**_r_* = 162.17	*F*(000) = 672
Triclinic, *P*1	*D*_x_ = 1.311 Mg m^−^^3^
*a* = 10.3961 (5) Å	Cu *K*α radiation, λ = 1.54184 Å
*b* = 10.8565 (6) Å	Cell parameters from 4341 reflections
*c* = 16.1431 (7) Å	θ = 4.4–71.4°
α = 84.704 (4)°	µ = 0.81 mm^−^^1^
β = 76.223 (4)°	*T* = 173 K
γ = 68.249 (5)°	Irregular, colourless
*V* = 1643.57 (16) Å^3^	0.22 × 0.16 × 0.10 mm

### Data collection

**Table:**

Agilent Eos Gemini diffractometer	6209 independent reflections
Radiation source: Enhance (Cu) X-ray Source	5042 reflections with *I* > 2σ(*I*)
Graphite monochromator	*R*_int_ = 0.019
Detector resolution: 16.0416 pixels mm^-1^	θ_max_ = 71.3°, θ_min_ = 4.4°
ω scans	*h* = −12→6
Absorption correction: multi-scan (*CrysAlis PRO* and *CrysAlis RED*, Agilent (2012).	*k* = −13→12
*T*_min_ = 0.881, *T*_max_ = 1.000	*l* = −19→18
11343 measured reflections	

### Refinement

**Table:**

Refinement on *F*^2^	Primary atom site location: structure-invariant direct methods
Least-squares matrix: full	Hydrogen site location: mixed
*R*[*F*^2^ > 2σ(*F*^2^)] = 0.044	H atoms treated by a mixture of independent and constrained refinement
*wR*(*F*^2^) = 0.122	*w* = 1/[σ^2^(*F*_o_^2^) + (0.0568*P*)^2^ + 0.3619*P*] where *P* = (*F*_o_^2^ + 2*F*_c_^2^)/3
*S* = 1.03	(Δ/σ)_max_ < 0.001
6209 reflections	Δρ_max_ = 0.26 e Å^−^^3^
449 parameters	Δρ_min_ = −0.22 e Å^−^^3^
0 restraints	

### Special details

**Table:**

Geometry. All esds (except the esd in the dihedral angle between two l.s. planes) are estimated using the full covariance matrix. The cell esds are taken into account individually in the estimation of esds in distances, angles and torsion angles; correlations between esds in cell parameters are only used when they are defined by crystal symmetry. An approximate (isotropic) treatment of cell esds is used for estimating esds involving l.s. planes.

### Fractional atomic coordinates and isotropic or equivalent isotropic displacement parameters (Å^2^)

**Table:**

	*x*	*y*	*z*	*U*_iso_*/*U*_eq_	
F1A	0.91461 (15)	0.91870 (11)	0.36091 (9)	0.0741 (4)	
N1A	0.86295 (14)	0.36783 (13)	0.29541 (9)	0.0411 (3)	
N2A	0.89322 (16)	0.23574 (14)	0.30565 (10)	0.0464 (3)	
H2A	0.850 (3)	0.194 (3)	0.2780 (16)	0.085 (8)*	
C1A	0.93710 (16)	0.40020 (15)	0.34199 (9)	0.0368 (3)	
C2A	1.01500 (19)	0.28686 (17)	0.38175 (11)	0.0452 (4)	
H2AA	1.0764	0.2810	0.4183	0.054*	
C3A	0.9839 (2)	0.18559 (18)	0.35684 (12)	0.0493 (4)	
H3A	1.0208	0.0952	0.3733	0.059*	
C4A	0.92972 (16)	0.53758 (15)	0.34570 (9)	0.0357 (3)	
C5A	0.81711 (18)	0.64507 (17)	0.32574 (11)	0.0436 (4)	
H5A	0.7430	0.6299	0.3085	0.052*	
C6A	0.8112 (2)	0.77360 (17)	0.33054 (12)	0.0512 (4)	
H6A	0.7345	0.8467	0.3164	0.061*	
C7A	0.9187 (2)	0.79295 (17)	0.35624 (12)	0.0499 (4)	
C8A	1.0311 (2)	0.69078 (19)	0.37737 (12)	0.0521 (4)	
H8A	1.1037	0.7075	0.3954	0.063*	
C9A	1.03655 (19)	0.56220 (17)	0.37178 (11)	0.0455 (4)	
H9A	1.1140	0.4899	0.3859	0.055*	
F1B	0.3343 (2)	1.06776 (13)	0.43105 (13)	0.1157 (6)	
N1B	0.45578 (15)	0.46841 (13)	0.33291 (8)	0.0390 (3)	
H1B	0.500 (3)	0.495 (2)	0.2756 (17)	0.088 (8)*	
N2B	0.45026 (16)	0.34515 (13)	0.34547 (9)	0.0451 (3)	
C1B	0.39261 (16)	0.54133 (15)	0.40414 (9)	0.0336 (3)	
C2B	0.34397 (18)	0.46175 (16)	0.46561 (10)	0.0425 (4)	
H2B	0.2948	0.4846	0.5229	0.051*	
C3B	0.3821 (2)	0.34208 (16)	0.42597 (11)	0.0473 (4)	
H3B	0.3623	0.2674	0.4527	0.057*	
C4B	0.37974 (16)	0.67949 (15)	0.40913 (10)	0.0357 (3)	
C5B	0.3388 (2)	0.73875 (18)	0.48847 (12)	0.0514 (4)	
H5B	0.3209	0.6886	0.5384	0.062*	
C6B	0.3238 (2)	0.8687 (2)	0.49627 (16)	0.0677 (6)	
H6B	0.2963	0.9084	0.5509	0.081*	
C7B	0.3493 (3)	0.93936 (19)	0.42403 (18)	0.0711 (6)	
C8B	0.3899 (3)	0.8856 (2)	0.34485 (17)	0.0778 (7)	
H8B	0.4076	0.9369	0.2955	0.093*	
C9B	0.4048 (2)	0.75479 (19)	0.33745 (13)	0.0581 (5)	
H9B	0.4327	0.7162	0.2825	0.070*	
F1C	0.06409 (14)	0.15006 (18)	0.11668 (9)	0.0902 (5)	
N1C	0.62673 (16)	0.14637 (14)	0.21875 (9)	0.0428 (3)	
H1C	0.560 (3)	0.214 (3)	0.2606 (19)	0.106 (9)*	
N2C	0.76704 (16)	0.10753 (14)	0.21687 (10)	0.0496 (4)	
C1C	0.60268 (18)	0.07149 (15)	0.16611 (10)	0.0397 (3)	
C2C	0.7333 (2)	−0.01966 (17)	0.12914 (12)	0.0485 (4)	
H2C	0.7522	−0.0865	0.0891	0.058*	
C3C	0.8310 (2)	0.00694 (18)	0.16274 (13)	0.0521 (4)	
H3C	0.9306	−0.0405	0.1489	0.063*	
C4C	0.46026 (18)	0.09391 (16)	0.15406 (10)	0.0402 (4)	
C5C	0.4371 (2)	−0.0033 (2)	0.11510 (12)	0.0559 (5)	
H5C	0.5139	−0.0835	0.0969	0.067*	
C6C	0.3038 (3)	0.0154 (2)	0.10251 (14)	0.0673 (6)	
H6C	0.2885	−0.0511	0.0761	0.081*	
C7C	0.1955 (2)	0.1309 (3)	0.12873 (12)	0.0602 (5)	
C8C	0.2129 (2)	0.2291 (2)	0.16722 (12)	0.0581 (5)	
H8C	0.1350	0.3088	0.1849	0.070*	
C9C	0.34629 (18)	0.21022 (18)	0.17996 (11)	0.0470 (4)	
H9C	0.3597	0.2776	0.2067	0.056*	
F1D	0.00377 (12)	0.72341 (13)	0.04635 (8)	0.0704 (3)	
N1D	0.59139 (15)	0.52634 (14)	0.16671 (8)	0.0429 (3)	
N2D	0.72929 (16)	0.51037 (17)	0.15930 (10)	0.0501 (4)	
H2D	0.778 (3)	0.459 (3)	0.206 (2)	0.114 (10)*	
C1D	0.55077 (17)	0.59754 (16)	0.09872 (9)	0.0388 (3)	
C2D	0.6648 (2)	0.62687 (19)	0.04769 (11)	0.0503 (4)	
H2DA	0.6660	0.6760	−0.0042	0.060*	
C3D	0.7748 (2)	0.5694 (2)	0.08865 (12)	0.0562 (5)	
H3D	0.8677	0.5715	0.0697	0.067*	
C4D	0.40598 (17)	0.63199 (15)	0.08592 (10)	0.0380 (3)	
C5D	0.29879 (19)	0.61209 (19)	0.14987 (11)	0.0507 (4)	
H5D	0.3192	0.5763	0.2032	0.061*	
C6D	0.1633 (2)	0.6436 (2)	0.13699 (12)	0.0570 (5)	
H6D	0.0906	0.6300	0.1808	0.068*	
C7D	0.13651 (18)	0.69495 (18)	0.05921 (12)	0.0489 (4)	
C8D	0.2375 (2)	0.71705 (17)	−0.00511 (11)	0.0492 (4)	
H8D	0.2156	0.7536	−0.0580	0.059*	
C9D	0.37280 (19)	0.68496 (16)	0.00855 (10)	0.0436 (4)	
H9D	0.4443	0.6994	−0.0358	0.052*	

### Atomic displacement parameters (Å^2^)

**Table:**

	*U*^11^	*U*^22^	*U*^33^	*U*^12^	*U*^13^	*U*^23^
F1A	0.0908 (9)	0.0394 (6)	0.0995 (10)	−0.0298 (6)	−0.0218 (7)	−0.0071 (6)
N1A	0.0438 (7)	0.0414 (7)	0.0421 (7)	−0.0192 (6)	−0.0082 (6)	−0.0062 (6)
N2A	0.0523 (8)	0.0424 (8)	0.0506 (8)	−0.0241 (7)	−0.0091 (7)	−0.0069 (6)
C1A	0.0385 (8)	0.0405 (8)	0.0324 (7)	−0.0167 (6)	−0.0045 (6)	−0.0034 (6)
C2A	0.0554 (10)	0.0409 (9)	0.0451 (9)	−0.0210 (8)	−0.0162 (8)	0.0013 (7)
C3A	0.0580 (10)	0.0389 (9)	0.0532 (10)	−0.0202 (8)	−0.0123 (8)	0.0006 (7)
C4A	0.0391 (8)	0.0377 (8)	0.0299 (7)	−0.0159 (6)	−0.0023 (6)	−0.0026 (6)
C5A	0.0448 (9)	0.0439 (9)	0.0440 (9)	−0.0180 (7)	−0.0096 (7)	−0.0004 (7)
C6A	0.0542 (10)	0.0386 (9)	0.0566 (11)	−0.0124 (8)	−0.0119 (8)	0.0013 (8)
C7A	0.0627 (11)	0.0362 (9)	0.0532 (10)	−0.0229 (8)	−0.0068 (8)	−0.0061 (7)
C8A	0.0553 (10)	0.0502 (10)	0.0607 (11)	−0.0263 (8)	−0.0163 (9)	−0.0072 (8)
C9A	0.0469 (9)	0.0413 (9)	0.0521 (10)	−0.0164 (7)	−0.0159 (8)	−0.0032 (7)
F1B	0.1591 (16)	0.0346 (7)	0.1658 (17)	−0.0410 (9)	−0.0453 (13)	−0.0091 (8)
N1B	0.0529 (8)	0.0327 (6)	0.0319 (7)	−0.0170 (6)	−0.0067 (6)	−0.0028 (5)
N2B	0.0587 (9)	0.0327 (7)	0.0467 (8)	−0.0178 (6)	−0.0127 (7)	−0.0054 (6)
C1B	0.0371 (8)	0.0331 (7)	0.0321 (7)	−0.0139 (6)	−0.0083 (6)	0.0000 (6)
C2B	0.0522 (10)	0.0391 (8)	0.0366 (8)	−0.0214 (7)	−0.0028 (7)	0.0004 (6)
C3B	0.0612 (11)	0.0357 (8)	0.0517 (10)	−0.0265 (8)	−0.0122 (8)	0.0046 (7)
C4B	0.0358 (8)	0.0323 (7)	0.0391 (8)	−0.0128 (6)	−0.0071 (6)	−0.0011 (6)
C5B	0.0625 (11)	0.0395 (9)	0.0488 (10)	−0.0170 (8)	−0.0049 (8)	−0.0092 (7)
C6B	0.0804 (15)	0.0426 (10)	0.0767 (14)	−0.0170 (10)	−0.0115 (11)	−0.0216 (10)
C7B	0.0859 (15)	0.0292 (9)	0.1048 (18)	−0.0217 (9)	−0.0287 (14)	−0.0076 (10)
C8B	0.119 (2)	0.0464 (11)	0.0788 (16)	−0.0452 (13)	−0.0219 (14)	0.0153 (11)
C9B	0.0885 (15)	0.0437 (10)	0.0467 (10)	−0.0342 (10)	−0.0078 (10)	0.0029 (8)
F1C	0.0624 (8)	0.1541 (15)	0.0731 (9)	−0.0566 (9)	−0.0189 (7)	−0.0068 (9)
N1C	0.0485 (8)	0.0366 (7)	0.0464 (8)	−0.0168 (6)	−0.0111 (6)	−0.0065 (6)
N2C	0.0511 (8)	0.0389 (7)	0.0637 (9)	−0.0168 (6)	−0.0203 (7)	−0.0021 (7)
C1C	0.0533 (9)	0.0337 (8)	0.0354 (8)	−0.0190 (7)	−0.0100 (7)	−0.0012 (6)
C2C	0.0563 (10)	0.0386 (9)	0.0508 (10)	−0.0135 (8)	−0.0154 (8)	−0.0082 (7)
C3C	0.0493 (10)	0.0410 (9)	0.0638 (11)	−0.0101 (8)	−0.0171 (9)	−0.0031 (8)
C4C	0.0501 (9)	0.0444 (9)	0.0302 (7)	−0.0236 (7)	−0.0059 (7)	0.0001 (6)
C5C	0.0648 (12)	0.0559 (11)	0.0554 (11)	−0.0272 (9)	−0.0157 (9)	−0.0112 (9)
C6C	0.0776 (15)	0.0851 (16)	0.0592 (12)	−0.0482 (13)	−0.0175 (11)	−0.0111 (11)
C7C	0.0541 (11)	0.0995 (17)	0.0409 (9)	−0.0436 (11)	−0.0104 (8)	0.0011 (10)
C8C	0.0469 (10)	0.0782 (14)	0.0454 (10)	−0.0226 (9)	−0.0005 (8)	−0.0070 (9)
C9C	0.0480 (9)	0.0548 (10)	0.0393 (9)	−0.0221 (8)	−0.0035 (7)	−0.0069 (7)
F1D	0.0458 (6)	0.0818 (9)	0.0781 (8)	−0.0133 (6)	−0.0200 (6)	0.0014 (7)
N1D	0.0442 (7)	0.0503 (8)	0.0321 (7)	−0.0153 (6)	−0.0061 (6)	−0.0040 (6)
N2D	0.0475 (8)	0.0618 (10)	0.0423 (8)	−0.0202 (7)	−0.0096 (7)	−0.0047 (7)
C1D	0.0461 (9)	0.0385 (8)	0.0300 (7)	−0.0159 (7)	−0.0027 (6)	−0.0039 (6)
C2D	0.0540 (10)	0.0567 (11)	0.0419 (9)	−0.0266 (9)	−0.0046 (8)	0.0042 (8)
C3D	0.0503 (10)	0.0704 (13)	0.0528 (11)	−0.0311 (9)	−0.0039 (8)	−0.0038 (9)
C4D	0.0449 (8)	0.0335 (7)	0.0323 (7)	−0.0130 (6)	−0.0036 (6)	−0.0025 (6)
C5D	0.0481 (10)	0.0642 (11)	0.0343 (8)	−0.0183 (8)	−0.0049 (7)	0.0061 (8)
C6D	0.0452 (10)	0.0727 (13)	0.0454 (10)	−0.0199 (9)	0.0016 (8)	0.0012 (9)
C7D	0.0409 (9)	0.0461 (9)	0.0546 (10)	−0.0078 (7)	−0.0125 (8)	−0.0047 (8)
C8D	0.0583 (11)	0.0444 (9)	0.0420 (9)	−0.0140 (8)	−0.0151 (8)	0.0044 (7)
C9D	0.0512 (9)	0.0420 (9)	0.0359 (8)	−0.0175 (7)	−0.0059 (7)	0.0023 (7)

### Geometric parameters (Å, º)

**Table:**

F1A—C7A	1.3584 (19)	F1C—C7C	1.362 (2)
N1A—N2A	1.3525 (19)	N1C—H1C	0.98 (3)
N1A—C1A	1.345 (2)	N1C—N2C	1.353 (2)
N2A—H2A	0.94 (3)	N1C—C1C	1.351 (2)
N2A—C3A	1.333 (2)	N2C—C3C	1.326 (2)
C1A—C2A	1.395 (2)	C1C—C2C	1.381 (2)
C1A—C4A	1.471 (2)	C1C—C4C	1.466 (2)
C2A—H2AA	0.9500	C2C—H2C	0.9500
C2A—C3A	1.372 (2)	C2C—C3C	1.384 (3)
C3A—H3A	0.9500	C3C—H3C	0.9500
C4A—C5A	1.391 (2)	C4C—C5C	1.396 (2)
C4A—C9A	1.395 (2)	C4C—C9C	1.388 (2)
C5A—H5A	0.9500	C5C—H5C	0.9500
C5A—C6A	1.382 (2)	C5C—C6C	1.386 (3)
C6A—H6A	0.9500	C6C—H6C	0.9500
C6A—C7A	1.370 (3)	C6C—C7C	1.357 (3)
C7A—C8A	1.368 (3)	C7C—C8C	1.369 (3)
C8A—H8A	0.9500	C8C—H8C	0.9500
C8A—C9A	1.387 (2)	C8C—C9C	1.387 (3)
C9A—H9A	0.9500	C9C—H9C	0.9500
F1B—C7B	1.356 (2)	F1D—C7D	1.361 (2)
N1B—H1B	1.00 (3)	N1D—N2D	1.356 (2)
N1B—N2B	1.3551 (18)	N1D—C1D	1.343 (2)
N1B—C1B	1.343 (2)	N2D—H2D	1.02 (3)
N2B—C3B	1.328 (2)	N2D—C3D	1.329 (2)
C1B—C2B	1.383 (2)	C1D—C2D	1.397 (2)
C1B—C4B	1.464 (2)	C1D—C4D	1.471 (2)
C2B—H2B	0.9500	C2D—H2DA	0.9500
C2B—C3B	1.380 (2)	C2D—C3D	1.375 (3)
C3B—H3B	0.9500	C3D—H3D	0.9500
C4B—C5B	1.388 (2)	C4D—C5D	1.394 (2)
C4B—C9B	1.384 (2)	C4D—C9D	1.391 (2)
C5B—H5B	0.9500	C5D—H5D	0.9500
C5B—C6B	1.375 (3)	C5D—C6D	1.383 (3)
C6B—H6B	0.9500	C6D—H6D	0.9500
C6B—C7B	1.362 (3)	C6D—C7D	1.374 (3)
C7B—C8B	1.361 (3)	C7D—C8D	1.364 (3)
C8B—H8B	0.9500	C8D—H8D	0.9500
C8B—C9B	1.383 (3)	C8D—C9D	1.385 (2)
C9B—H9B	0.9500	C9D—H9D	0.9500
			
C1A—N1A—N2A	106.39 (13)	N2C—N1C—H1C	118.1 (16)
N1A—N2A—H2A	118.9 (16)	C1C—N1C—H1C	130.5 (17)
C3A—N2A—N1A	110.49 (14)	C1C—N1C—N2C	110.98 (14)
C3A—N2A—H2A	130.6 (16)	C3C—N2C—N1C	105.89 (14)
N1A—C1A—C2A	109.52 (14)	N1C—C1C—C2C	106.74 (15)
N1A—C1A—C4A	121.34 (14)	N1C—C1C—C4C	122.62 (15)
C2A—C1A—C4A	129.14 (14)	C2C—C1C—C4C	130.62 (15)
C1A—C2A—H2AA	127.4	C1C—C2C—H2C	127.3
C3A—C2A—C1A	105.28 (15)	C1C—C2C—C3C	105.42 (15)
C3A—C2A—H2AA	127.4	C3C—C2C—H2C	127.3
N2A—C3A—C2A	108.33 (16)	N2C—C3C—C2C	110.97 (16)
N2A—C3A—H3A	125.8	N2C—C3C—H3C	124.5
C2A—C3A—H3A	125.8	C2C—C3C—H3C	124.5
C5A—C4A—C1A	121.94 (14)	C5C—C4C—C1C	119.78 (16)
C5A—C4A—C9A	118.47 (15)	C9C—C4C—C1C	121.88 (15)
C9A—C4A—C1A	119.57 (14)	C9C—C4C—C5C	118.33 (17)
C4A—C5A—H5A	119.4	C4C—C5C—H5C	119.5
C6A—C5A—C4A	121.12 (16)	C6C—C5C—C4C	121.1 (2)
C6A—C5A—H5A	119.4	C6C—C5C—H5C	119.5
C5A—C6A—H6A	120.8	C5C—C6C—H6C	120.7
C7A—C6A—C5A	118.34 (17)	C7C—C6C—C5C	118.55 (19)
C7A—C6A—H6A	120.8	C7C—C6C—H6C	120.7
F1A—C7A—C6A	118.98 (17)	F1C—C7C—C8C	118.5 (2)
F1A—C7A—C8A	118.17 (17)	C6C—C7C—F1C	118.9 (2)
C8A—C7A—C6A	122.85 (16)	C6C—C7C—C8C	122.57 (18)
C7A—C8A—H8A	120.8	C7C—C8C—H8C	120.6
C7A—C8A—C9A	118.35 (17)	C7C—C8C—C9C	118.84 (19)
C9A—C8A—H8A	120.8	C9C—C8C—H8C	120.6
C4A—C9A—H9A	119.6	C4C—C9C—H9C	119.7
C8A—C9A—C4A	120.86 (16)	C8C—C9C—C4C	120.64 (17)
C8A—C9A—H9A	119.6	C8C—C9C—H9C	119.7
N2B—N1B—H1B	120.0 (15)	C1D—N1D—N2D	106.73 (14)
C1B—N1B—H1B	128.9 (15)	N1D—N2D—H2D	117.9 (17)
C1B—N1B—N2B	111.08 (13)	C3D—N2D—N1D	110.18 (15)
C3B—N2B—N1B	105.72 (13)	C3D—N2D—H2D	131.9 (17)
N1B—C1B—C2B	106.85 (14)	N1D—C1D—C2D	109.36 (15)
N1B—C1B—C4B	123.31 (13)	N1D—C1D—C4D	121.20 (14)
C2B—C1B—C4B	129.83 (14)	C2D—C1D—C4D	129.44 (15)
C1B—C2B—H2B	127.3	C1D—C2D—H2DA	127.4
C3B—C2B—C1B	105.37 (14)	C3D—C2D—C1D	105.16 (16)
C3B—C2B—H2B	127.3	C3D—C2D—H2DA	127.4
N2B—C3B—C2B	110.99 (14)	N2D—C3D—C2D	108.57 (16)
N2B—C3B—H3B	124.5	N2D—C3D—H3D	125.7
C2B—C3B—H3B	124.5	C2D—C3D—H3D	125.7
C5B—C4B—C1B	119.17 (14)	C5D—C4D—C1D	121.57 (15)
C9B—C4B—C1B	122.57 (15)	C9D—C4D—C1D	120.35 (14)
C9B—C4B—C5B	118.26 (16)	C9D—C4D—C5D	118.08 (16)
C4B—C5B—H5B	119.4	C4D—C5D—H5D	119.4
C6B—C5B—C4B	121.24 (18)	C6D—C5D—C4D	121.17 (16)
C6B—C5B—H5B	119.4	C6D—C5D—H5D	119.4
C5B—C6B—H6B	120.7	C5D—C6D—H6D	120.8
C7B—C6B—C5B	118.6 (2)	C7D—C6D—C5D	118.35 (17)
C7B—C6B—H6B	120.7	C7D—C6D—H6D	120.8
F1B—C7B—C6B	119.0 (2)	F1D—C7D—C6D	118.09 (17)
F1B—C7B—C8B	118.7 (2)	F1D—C7D—C8D	119.25 (16)
C8B—C7B—C6B	122.33 (18)	C8D—C7D—C6D	122.65 (17)
C7B—C8B—H8B	120.6	C7D—C8D—H8D	120.8
C7B—C8B—C9B	118.8 (2)	C7D—C8D—C9D	118.44 (16)
C9B—C8B—H8B	120.6	C9D—C8D—H8D	120.8
C4B—C9B—H9B	119.6	C4D—C9D—H9D	119.3
C8B—C9B—C4B	120.79 (19)	C8D—C9D—C4D	121.31 (16)
C8B—C9B—H9B	119.6	C8D—C9D—H9D	119.3
			
F1A—C7A—C8A—C9A	−179.30 (16)	F1C—C7C—C8C—C9C	179.81 (17)
N1A—N2A—C3A—C2A	0.2 (2)	N1C—N2C—C3C—C2C	0.2 (2)
N1A—C1A—C2A—C3A	−0.03 (19)	N1C—C1C—C2C—C3C	−0.09 (19)
N1A—C1A—C4A—C5A	20.9 (2)	N1C—C1C—C4C—C5C	−165.31 (16)
N1A—C1A—C4A—C9A	−160.61 (15)	N1C—C1C—C4C—C9C	15.0 (2)
N2A—N1A—C1A—C2A	0.12 (18)	N2C—N1C—C1C—C2C	0.23 (19)
N2A—N1A—C1A—C4A	179.93 (13)	N2C—N1C—C1C—C4C	−178.51 (14)
C1A—N1A—N2A—C3A	−0.17 (19)	C1C—N1C—N2C—C3C	−0.28 (19)
C1A—C2A—C3A—N2A	−0.1 (2)	C1C—C2C—C3C—N2C	−0.1 (2)
C1A—C4A—C5A—C6A	179.25 (15)	C1C—C4C—C5C—C6C	−179.71 (17)
C1A—C4A—C9A—C8A	−178.84 (16)	C1C—C4C—C9C—C8C	179.57 (16)
C2A—C1A—C4A—C5A	−159.34 (17)	C2C—C1C—C4C—C5C	16.3 (3)
C2A—C1A—C4A—C9A	19.2 (2)	C2C—C1C—C4C—C9C	−163.36 (18)
C4A—C1A—C2A—C3A	−179.82 (16)	C4C—C1C—C2C—C3C	178.51 (17)
C4A—C5A—C6A—C7A	−0.5 (3)	C4C—C5C—C6C—C7C	0.2 (3)
C5A—C4A—C9A—C8A	−0.3 (2)	C5C—C4C—C9C—C8C	−0.1 (3)
C5A—C6A—C7A—F1A	179.73 (16)	C5C—C6C—C7C—F1C	−179.94 (18)
C5A—C6A—C7A—C8A	−0.1 (3)	C5C—C6C—C7C—C8C	−0.2 (3)
C6A—C7A—C8A—C9A	0.5 (3)	C6C—C7C—C8C—C9C	0.0 (3)
C7A—C8A—C9A—C4A	−0.3 (3)	C7C—C8C—C9C—C4C	0.1 (3)
C9A—C4A—C5A—C6A	0.7 (2)	C9C—C4C—C5C—C6C	0.0 (3)
F1B—C7B—C8B—C9B	−179.8 (2)	F1D—C7D—C8D—C9D	−178.71 (16)
N1B—N2B—C3B—C2B	−0.2 (2)	N1D—N2D—C3D—C2D	0.2 (2)
N1B—C1B—C2B—C3B	−0.10 (19)	N1D—C1D—C2D—C3D	0.1 (2)
N1B—C1B—C4B—C5B	−167.30 (16)	N1D—C1D—C4D—C5D	10.9 (2)
N1B—C1B—C4B—C9B	13.7 (3)	N1D—C1D—C4D—C9D	−168.65 (15)
N2B—N1B—C1B—C2B	0.01 (18)	N2D—N1D—C1D—C2D	0.01 (19)
N2B—N1B—C1B—C4B	−178.70 (13)	N2D—N1D—C1D—C4D	179.25 (14)
C1B—N1B—N2B—C3B	0.09 (19)	C1D—N1D—N2D—C3D	−0.1 (2)
C1B—C2B—C3B—N2B	0.2 (2)	C1D—C2D—C3D—N2D	−0.2 (2)
C1B—C4B—C5B—C6B	−179.28 (18)	C1D—C4D—C5D—C6D	−179.36 (17)
C1B—C4B—C9B—C8B	179.22 (19)	C1D—C4D—C9D—C8D	179.45 (15)
C2B—C1B—C4B—C5B	14.3 (3)	C2D—C1D—C4D—C5D	−170.03 (18)
C2B—C1B—C4B—C9B	−164.73 (18)	C2D—C1D—C4D—C9D	10.4 (3)
C4B—C1B—C2B—C3B	178.49 (16)	C4D—C1D—C2D—C3D	−179.03 (16)
C4B—C5B—C6B—C7B	0.3 (3)	C4D—C5D—C6D—C7D	0.1 (3)
C5B—C4B—C9B—C8B	0.2 (3)	C5D—C4D—C9D—C8D	−0.1 (2)
C5B—C6B—C7B—F1B	179.7 (2)	C5D—C6D—C7D—F1D	178.79 (17)
C5B—C6B—C7B—C8B	−0.4 (4)	C5D—C6D—C7D—C8D	−0.6 (3)
C6B—C7B—C8B—C9B	0.4 (4)	C6D—C7D—C8D—C9D	0.7 (3)
C7B—C8B—C9B—C4B	−0.3 (4)	C7D—C8D—C9D—C4D	−0.3 (3)
C9B—C4B—C5B—C6B	−0.2 (3)	C9D—C4D—C5D—C6D	0.2 (3)

### Hydrogen-bond geometry (Å, º)

**Table:**

*D*—H···*A*	*D*—H	H···*A*	*D*···*A*	*D*—H···*A*
N2*A*—H2*A*···N2*C*	0.94 (3)	1.94 (3)	2.886 (2)	177 (2)
C3*A*—H3*A*···F1*A*^i^	0.95	2.58	3.226 (2)	125
N1*B*—H1*B*···N1*D*	1.00 (3)	1.86 (3)	2.8506 (19)	175 (2)
C3*B*—H3*B*···F1*B*^i^	0.95	2.36	3.187 (2)	145
C6*B*—H6*B*···F1*A*^ii^	0.95	2.51	3.287 (3)	139
N1*C*—H1*C*···N2*B*	0.98 (3)	1.90 (3)	2.881 (2)	173 (2)
N2*D*—H2*D*···N1*A*	1.02 (3)	1.87 (3)	2.896 (2)	178 (3)
C3*D*—H3*D*···F1*D*^iii^	0.95	2.49	3.301 (2)	143

Symmetry codes: (i) *x*, *y*−1, *z*; (ii) −*x*+1, −*y*+2, −*z*+1; (iii) *x*+1, *y*, *z*.
