# Supplementary figures and images for: Two tautomers in the same crystal: 3-(4-fluoro­phen­yl)-1H-pyrazole and 5-(4-fluoro­phen­yl)-1H-pyrazole
Source: Acta Crystallogr Sect E Struct Rep Online. 2014 Aug 1;70(Pt 9):o949–50. doi: 10.1107/S160053681401695X (PMC4186127; doi:10.1107/S160053681401695X)

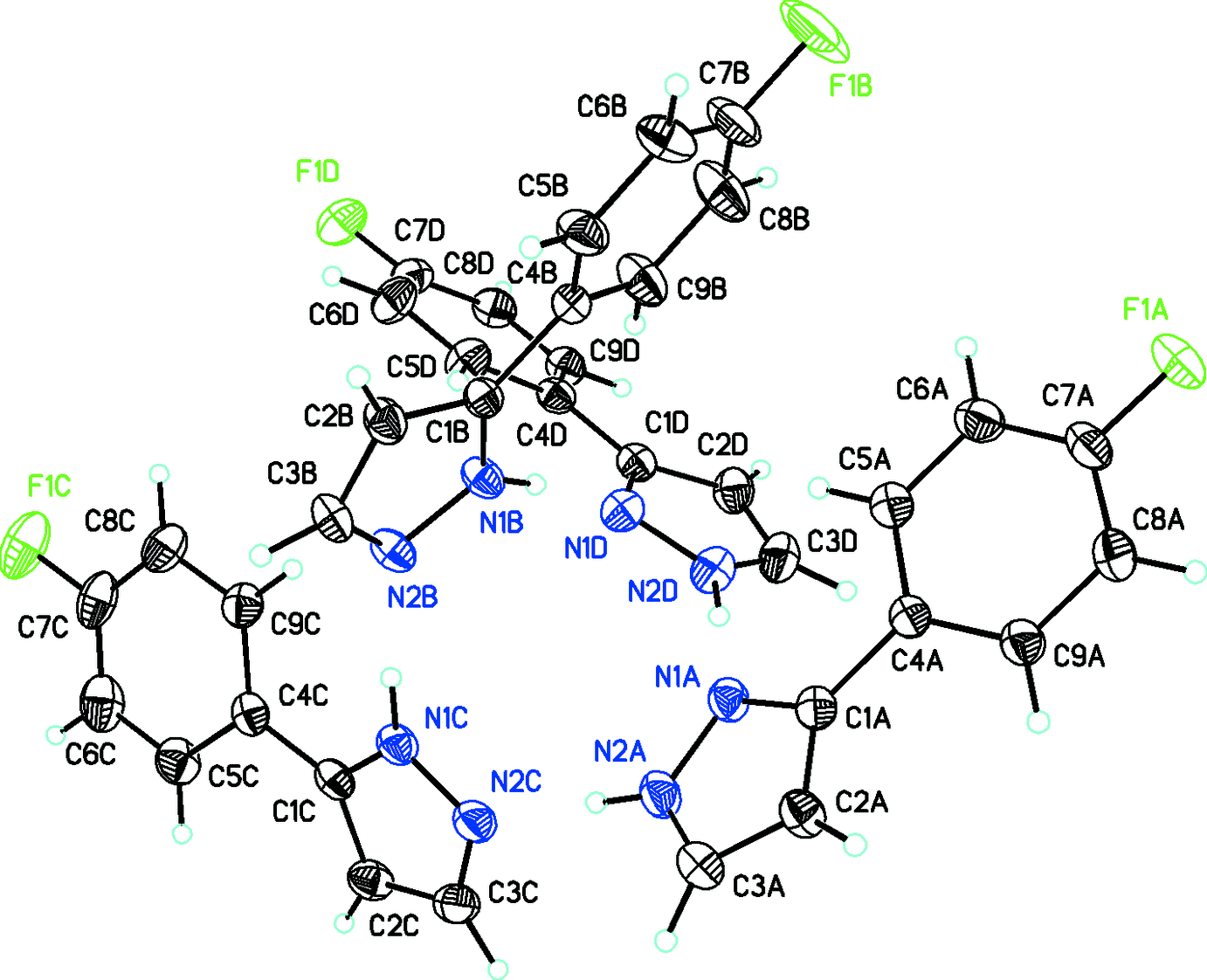

Supplement: Supplementary file 4 [file e-70-0o949-fig1.tif]

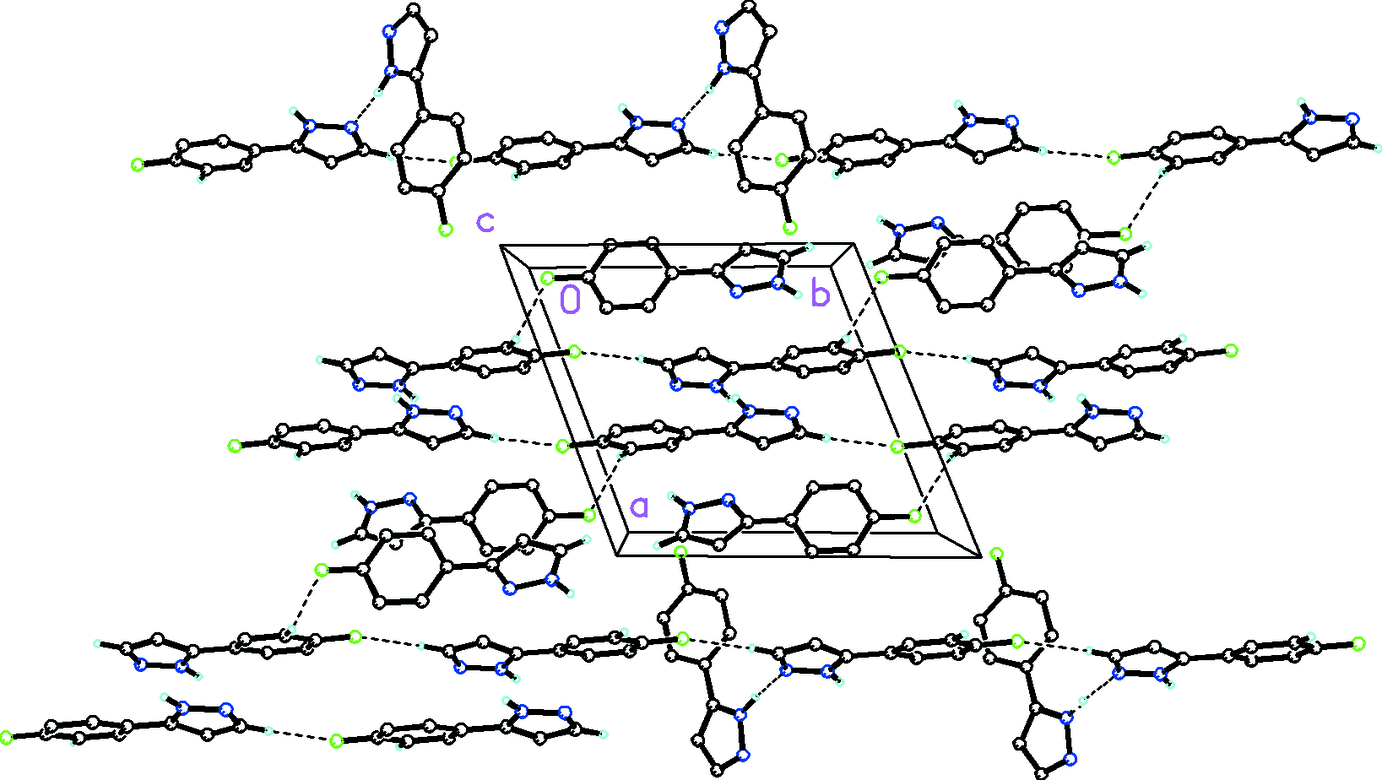

Supplement: Supplementary file 5 [file e-70-0o949-fig2.tif]

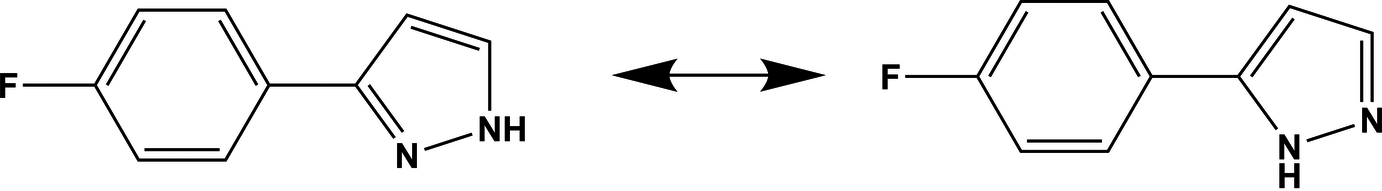

Supplement: Supplementary file 6 [file e-70-0o949-fig3.tif]
